# Induction of Atrial Fibrillation by Neutrophils Critically Depends on CD11b/CD18 Integrins

**DOI:** 10.1371/journal.pone.0089307

**Published:** 2014-02-18

**Authors:** Kai Friedrichs, Matti Adam, Lisa Remane, Martin Mollenhauer, Volker Rudolph, Tanja K. Rudolph, René P. Andrié, Florian Stöckigt, Jan W. Schrickel, Thorben Ravekes, Florian Deuschl, Georg Nickenig, Stephan Willems, Stephan Baldus, Anna Klinke

**Affiliations:** 1 Heart Center, University of Cologne, Cologne, Germany; 2 Cologne Cardiovascular Research Center, University of Cologne, Cologne, Germany; 3 Department of General and Interventional Cardiology, University Heart Center Hamburg, Hamburg, Germany; 4 Department of Medicine-Cardiology, University Hospital of Bonn, Bonn, Germany; 5 Department of Electrophysiology, University Heart Center Hamburg, Hamburg, Germany; University of Illinois at Chicago, United States of America

## Abstract

**Background:**

Recent observational clinical and *ex-vivo* studies suggest that inflammation and in particular leukocyte activation predisposes to atrial fibrillation (AF). However, whether local binding and extravasation of leukocytes into atrial myocardium is an essential prerequisite for the initiation and propagation of AF remains elusive. Here we investigated the role of atrial CD11b/CD18 mediated infiltration of polymorphonuclear neutrophils (PMN) for the susceptibility to AF.

**Methods and Results:**

C57bl/6J wildtype (WT) and CD11b/CD18 knock-out (CD11b^−/−^) mice were treated for 14 days with subcutaneous infusion of angiotensin II (Ang II), a known stimulus for PMN activation. Atria of Ang II-treated WT mice were characterized by increased PMN infiltration assessed in immunohistochemically stained sections. In contrast, atrial sections of CD11b^−/−^ mice lacked a significant increase in PMN infiltration upon Ang II infusion. PMN infiltration was accompanied by profoundly enhanced atrial fibrosis in Ang II treated WT as compared to CD11b^−/−^ mice. Upon *in-vivo* electrophysiological investigation, Ang II treatment significantly elevated the susceptibility for AF in WT mice if compared to vehicle treated animals given an increased number and increased duration of AF episodes. In contrast, animals deficient of CD11b/CD18 were entirely protected from AF induction. Likewise, epicardial activation mapping revealed decreased electrical conduction velocity in atria of Ang II treated WT mice, which was preserved in CD11b^−/−^ mice. In addition, atrial PMN infiltration was enhanced in atrial appendage sections of patients with persistent AF as compared to patients without AF.

**Conclusions:**

The current data critically link CD11b-integrin mediated atrial PMN infiltration to the formation of fibrosis, which promotes the initiation and propagation of AF. These findings not only reveal a mechanistic role of leukocytes in AF but also point towards a potential novel avenue of treatment in AF.

## Introduction

Atrial fibrillation (AF) stands out as the most prevalent human rhythm disorder. Atrial fibrillation is associated with an increased long-term risk of heart failure, remains a principal and common cause of stroke and doubles mortality [1]–[4]. Despite its prevalence and its contribution to morbidity and mortality, treatment strategies still remain scarce. Ion channel directed therapies as well as interventional strategies are most effective only in a subset of patients: whereas patients with non permanent, paroxysmal AF can be treated in the majority of cases, individuals with permanent AF in large part do not derive benefit from anti-arrhythmic and interventional therapy, respectively, calling for adjunct therapies [5], [6].

Therefore, a better understanding of the underlying pathophysiology is of foremost importance. An accumulating body of evidence suggests that atrial fibrosis plays a major role in the pathogenesis of atrial fibrillation: Increased deposition of interstitial matrix such as collagen I and III and fibronectin impedes atrial conduction, allowing for an increased electrical ectopy and reentry. Moreover, matrix turnover - by exposing cytokines, adhesion molecules and growth factors – propagates a proinflammatory milieu [7]–[9]. In fact, inflammation appears to be a critical confounder for structural remodeling of the atria and thus for the genesis of atrial fibrillation [10], [11]. Clinical studies support this view by revealing a predictive role of biomarkers such as C-reactive protein (CRP), interleukin (IL)-6 and tumor necrosis factor (TNF)-α with respect to AF occurrence, persistence, recurrence and left atrial dimensions. [12]–[22]. More so, clinical observations revealed leukocytes to be of critical significance for this disease in humans: Leukocytes were identified in atrial tissue of AF patients even without an underlying structural heart disease [11], and postoperative atrial leukocyte infiltration independently predicted postsurgery AF [23], [24]. Of note, enzyme systems stored in leukocytes such as myeloperoxidase (MPO) and matrix metalloproteinase (MMP)-2, enzymes known to accelerate tissue remodeling, were also predictive of AF burden and recurrence of this disease following interventional ablation [25], [26].

Activation and extravasation of leukocytes and in particular of polymorphonuclear neutrophils (PMN), the most abundant subset of leukocytes and the major constituents of the innate immune system, depend on the activation state of the local endothelium – which releases local cytokines and expresses adhesion molecules. As of at least similar importance, PMN adhesion critically relies on the expression of integrins on the leukocytés outer membrane: PMN express CD11b/CD18 integrins (Mac1), which allow binding to adhesion molecules like intercellular adhesion molecule-1 (ICAM-1) on the endothelial cell surface – the principal prerequisite for the leukocytés subsequent extravasation [27]–[29]. Notably, CD11b/CD18 integrins not only interact with cellular proteins, they also bind with high affinity to components of the extracellular matrix like fibrinogen/fibrin and collagen and to polysaccharides like heparan sulfates [30]–[32]. Furthermore, CD11b/CD18 takes central stage in PMN activation: Effector pathways downstream of CD11b/CD18 include NADPH-oxidase activation with concomitant formation of superoxide and release of granular proteins [33], [34]. Interestingly, it has been shown lately, that PMN release MPO to endothelial cells via a direct CD11b/CD18-integrin mediated intercellular link [35].

We observed recently, that MPO promotes fibrosis and thereby increases AF susceptibility [25]. However, to date a direct mechanistic link between AF and PMN localization within the atrial tissue has not been established. Here we tested the impact of CD11b/CD18 integrins on AF susceptibility in a murine model of AF.

## Methods

### Ethics Statement

All animal studies were approved by the local authorities: (Behörde für Soziales, Familie, Gesundheit und Verbraucherschutz, Fachabteilung Veterinärwesen und Lebensmittelsicherheit, Hamburg, G09/064 and Landesamt für Natur, Umwelt und Verbraucherschutz Nordrhein-Westfalen, 84-02.04.2012.A307) and the Universities of Hamburg and Cologne Animal Care and Use Committees. All surgical interventions were performed under isoflurane anaesthesia and buprenorphine analgesia to minimize suffering of animals.

Patient studies were approved by the local Ethics Committee (Hamburg) and were performed in accordance with the Declaration of Helsinki and with written informed consent.

### Animals and experimental design

Male C57bl/6J WT and CD11b/CD18-deficient (Itgam_tm1Myd/J, CD11b^−/−^) mice (8–10 weeks of age, Jackson Laboratory) were treated with either angiotensin II (Ang II, 1.3 ng/g/min) or vehicle via subcutaneously implanted osmotic minipumps (Alzet, model 1002) for 2 weeks. We did not observe any differences in mortality, wound infection or wound healing after minipump implantation in CD11b^−/−^ compared to WT mice. These findings were consistent with previous work that reported similar observations [36].

### Electrophysiological investigation

Mice were anaesthetized with isoflurane and placed in supine position on a heating pad. An octapolar electrophysiological catheter (1.1 F, Scisense) was inserted via the right jugular vein to the right atrium and ventricle. Surface ECG was analyzed under stable baseline conditions for at least 3 min. Heart rate, P wave duration, QRS duration and QTc interval were measured by successive evaluation of 10 RR complexes in the most distinguishable tracings. Electrophysiological investigation with induction of arrhythmias was performed as described previously [37]. Intracardiac atrial and ventricular recording and atrial stimulation maneuvers were performed using a CardioTek EPTracer (Biotronik). Bipolar electrograms were obtained from each electrode pair during the whole procedure. Programmed atrial stimulation was performed at pacing stimulus amplitudes of 1.0 and 2.0 mA with 7 stimuli fixed rate at S1S1 cycle length of 120 ms, 110 ms and 100 ms, respectively, with one short coupled extra stimulus with a 10 ms-stepwise S1S2 reduction starting at cycle length of 80 ms down to 10 ms. Atrial refractory period (ARP) was determined, which was defined as longest S1S2 with absent atrial response in the most representative intracardiac tracing. Atrial burst stimulation was performed for 1 sec (three times consecutively) at S1S1 stimulation cycle lengths starting at 50 ms with 10-ms stepwise reduction down to 10 ms at pacing stimulus amplitudes of 1.0 and 2.0 mA. Between these stimulation procedures, a 10-sec recovery period was maintained. Atrial fibrillation was defined by presence of rapid and fragmented atrial electrograms in combination with irregular AV-nodal conduction and ventricular rhythm with a duration of these atrial electrograms of more than one second [38]. Number of AF episodes and AF duration (last stimulus-spike to the first sinus-rhythm P wave) were analyzed.

Thereafter, blood was drawn from the caval vein into heparinized syringes, hearts were flushed with saline via left ventricular puncture and hearts were excised. Hearts were either fixed in 3.7% paraformaldehyde solution and embedded in paraffin, embedded in optimal cutting temperature compound (OCT) and frozen to −80°C or atria and ventricles were dissected and snap frozen in liquid nitrogen.

### Langendorff-perfused hearts and epicardial mapping

For investigation of myocardial conduction velocities and homogeneity of conduction, hearts were Langendorff-perfused and epicardial activation mapping (EAM) using a 36-electrode array (FlexMEA36, Multi Channel Systems, interelectrode distance: 300 µm) was performed [37]. For this, hearts were excorporated and dissected from surrounding tissue in ice-cold Krebs-Henseleit buffer. Following cannulation of the aorta, the heart was immersed in a water-jacketed chamber and further fixed on a moisturized support. Hearts were then retrogradely perfused in a Langendorff-apparatus (Radnoti Technologies Inc.) at constant pressure perfusion (80 mmHg, resulting in coronary flow between 2–2.5 ml/min). The perfusate composition was (in mM): NaCl 110, KCl 4.6, MgSO_4_ 1.2, CaCl 2, NaH_2_PO_4_ 2, NaHCO_3_ 25, glucose 8.3, Na-pyruvate 2 and gassed with carbogen (O_2_ 95%, CO_2_ 5%), pH, 7.35–7.45 at constantly 37°C. 36 unipolar electrograms were recorded from the epicardial surface of both atria with regard to a reference electrode in the water-bath. Electrograms were recorded using a computer assisted recording system (Multi Channel Systems) with a sampling rate of up to 25 kHz. Data were band-pass filtered (50 Hz), digitized with 12 bit and a range of 20 mV.

Activation maps were calculated from these data using Cardio 2D Software (version 2.0.3, Multi Channel Systems). The first derivative of each unipolar electrogram was evaluated and maximal negative dV/dt activation was defined as the time-point of maximum local activation. With regard to myocardial fiber orientation, longitudinal and transversal conduction velocities (CV) were evaluated by calculating latencies between two electrodes, divided by the interelectrode distance.

### Immunofluorescence analysis

OCT embedded samples were cut to 3 µm sections and fixed with 3.7% formaldehyde. Tissue was permeabilized with 0.1% Triton-X 100 and treated with primary antibodies to murine Ly6G (Hycult Biotechnology, 1∶40) and MPO (Thermo Scientific, 1∶100) or for human sections to MPO (Calbiochem, 1∶200) and Alexa-Fluor-conjugated secondary antibodies (Invitrogen, 1∶200). Nuclei were stained with DAPI. Images of MPO and PMN in atrial tissue were captured with a CCD camera mounted on a Leica DMLB microscope with IVision software. Number of PMN was counted in 4–5 fields of view (magnification ×40) per atrium.

### Determination of MPO levels in heart perfusates

To release MPO from its binding to the vascular endothelium of the coronary circulation, hearts of anaesthetized mice were explanted and immediately cannulated via the aorta. Hearts were rinsed retrogradely with 200 µl of PBS followed by perfusion with 2 ml of heparin solution (5 I.U./ml). The perfusate was concentrated by vacuum centrifugation to 40 µl and the MPO concentration was determined by ELISA following manufacturer's instructions (Hycult Biotechnology).

### Determination of MPO levels in atrial homogenates

Samples were homogenized in lysis buffer (20 mM Tris-HCl pH 7.5, 250 mM succrose, 20 mM ETDA, 3 mM EGTA, 0.1% Triton X-100, supplemented with 10× ETDA-free Protease Inhibitor Tablets and 10× PhoSTOP; Roche Diagnostics) using the Tissue Lyzer (Qiagen). Homogenates were centrifuged at 14,000 g (4°C, 10 min) and the supernatant was recovered. MPO was quantified using an ELISA (Hycult Biotechnology) according to the manufacture's instruction. Total protein amount in samples was assessed with BCA-protein assay (Pierce). MPO levels were related to total protein.

### Immunoblot

Hearts were explanted from anaesthetized mice, rinsed in ice cold PBS and atria were dissected from ventricles. The tissue was snap frozen in liquid nitrogen and stored at −80°C. Samples were homogenated in lysis buffer as described above. Homogenates were centrifuged at 14,000 g (4°C, 10 min) and the supernatant was recovered. Proteins were separated by SDS-PAGE and transferred to nitrocellulose membranes. After blocking with 5% nonfat milk in TBST (20 mM Tris-HCl pH 7.5, 137 mM NaCl, 0.1% (v/v) Tween 20), membranes were incubated with primary antibodies to ICAM-1 (1∶200, Santa Cruz Biotechnology), VCAM-1 (1∶200, Santa Cruz Biotechnology) or GAPDH (1∶2,500, Cell Signaling Technology), followed by horseradish peroxidase-conjugated secondary antibodies (1∶10,000, Vector Laboratories) and chemiluminescence signals were detected with a Fusion FX Advance (Vilber Lourmat) and analyzed densitometrically with Fusion-CAPT software (Vilber Lourmat).

### Determination of atrial fibrosis

Longitudinal sections (4 µm) of paraffin embedded hearts were prepared and stained with Trichrome stain following a standard protocol. The area in atrial sections, which was stained in light blue (excluding pericardium), was quantified using color threshold and planimetry with Keyence BZII Analyzer (Keyence) software.

### Patients with AF

Right atrial appendages were obtained from patients undergoing elective coronary artery bypass surgery, either from patients with persistent AF or without AF.

### Statistical analysis

Continuous variables were tested for normal distribution by using the Kolmogorov-Smirnoff test. Data are presented as mean ± SEM or as median (line) and interquartile range (box); whiskers indicate 5% and 95% percentiles. Statistical analysis was performed by one-way ANOVA followed by Bonferroni or LSD *post hoc* test for normally distributed data, or Kruskal-Wallis test with Mann-Whitney-U *post hoc* test, as appropriate. For comparison of two groups of non-normally distributed data, Mann-Whitney U test was used. A value of P<0.05 was considered statistically significant. All calculations were carried out by using SPSS Statistics 20 for Mac.

## Results

**Figure 1:**
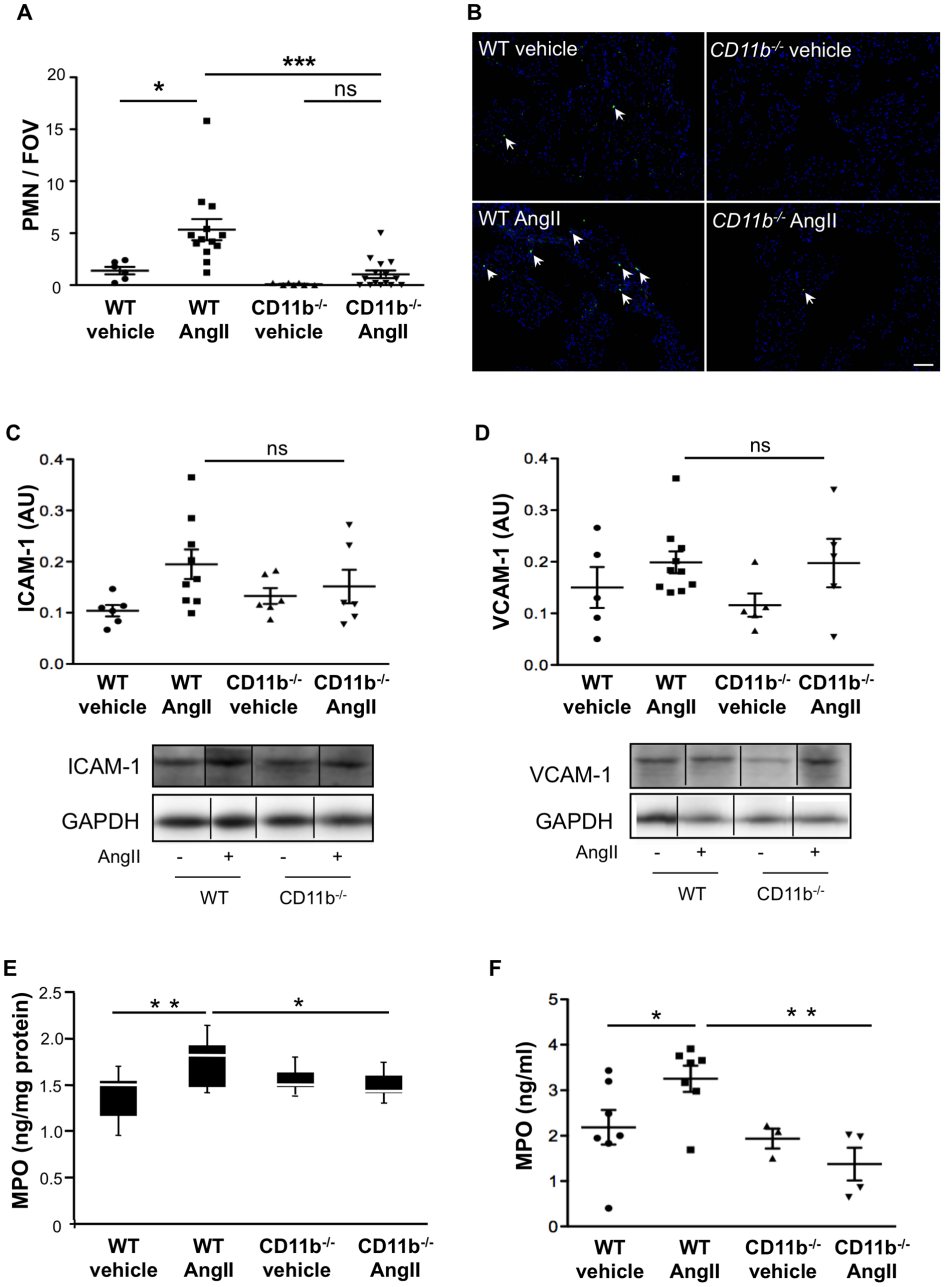
Atrial PMN infiltration and MPO accumulation was attenuated by CD11b-deficiency. **(A)** Number of MPO- and Ly6G-positive leukocytes in atrial sections of WT and CD11b^−/−^ mice upon vehicle or Ang II treatment was quantified in 4–5 FOVs per atrium (FOV  =  field of view, ×40). *  =  p<0.05, ***  =  p<0.001. **(B)** Representative images of immunofluorescence staining of PMN in mouse atrial tissue: blue  =  DAPI, red  =  Ly6G, green  =  MPO. Arrowheads indicate leukocytes. Scale bar  = 50 µm. **(C, D)** Protein expression of ICAM-1 and VCAM-1 in atrial tissue of WT and CD11b^−/−^ mice upon vehicle or Ang II infusion. Representative immunoblots are shown, where bands are spliced together as they were noncontinuous but were run on the same gel. **(E)** MPO in atrial tissue of WT and CD11b^−/−^ mice. *  =  p<0.05, **  =  p<0.01. **(F)** Amount of MPO deposition in the coronary circulation of WT and CD11b^−/−^ mice. *  =  p<0.05, **  =  p<0.01.

To induce leukocyte activation in wild type (WT) and CD11b/CD18 integrin-deficient (CD11b^−/−^) mice, Ang II was infused subcutaneously for 14 days by osmotic minipumps. Immunohistochemical analysis of atrial sections revealed increased atrial infiltration of PMN in WT mice (n = 13) as compared to vehicle treated animals (n = 6; p<0.05). This Ang II-dependent increase in PMN extravasation proved to be CD11b-dependent, since mice devoid of the integrin did not demonstrate any significant increase in extravascular deposition of PMN in the atria (Ang II: n = 15, vehicle: n = 7; p = 0.09) (**Fig. 1A, B**). Immunoblot analyses of the amount of endothelial CD11b/CD18 binding partners revealed that the protein amounts of ICAM-1 (WT ctrl, Ang II: n = 6, 9; CD11b^−/−^ ctrl, Ang II: 6, 6) and vascular cell adhesion molecule-1 (VCAM-1) (WT ctrl, Ang II: n = 5, 10; CD11b^−/−^ ctrl, Ang II: n = 5, 5) were slightly enhanced following Ang II application as compared to untreated animals, but were not different between Ang II treated WT and Ang II treated CD11b^−/−^ mice (ICAM-1 p = 0.12; VCAM-1 p = 0.98) (**Fig. 1C, D**). Analysis of cardiac MPO deposition in the different treatment groups revealed significantly lower MPO concentrations in atrial homogenates of CD11b^−/−^ mice upon Ang II treatment (n = 5) as compared to Ang II treated WT mice (n = 11, p<0.05) (**Fig. 1E**). Likewise, the amount of endothelial bound MPO within the coronary vasculature was markedly lower in Ang II treated CD11b^−/−^ (n = 4) as compared to Ang II treated WT mice (n = 7; p<0.01), thereby also indicating a decrease in cardiac MPO accumulation due to CD11b/CD18 deficiency (**Fig. 1F**).

**Figure 2:**
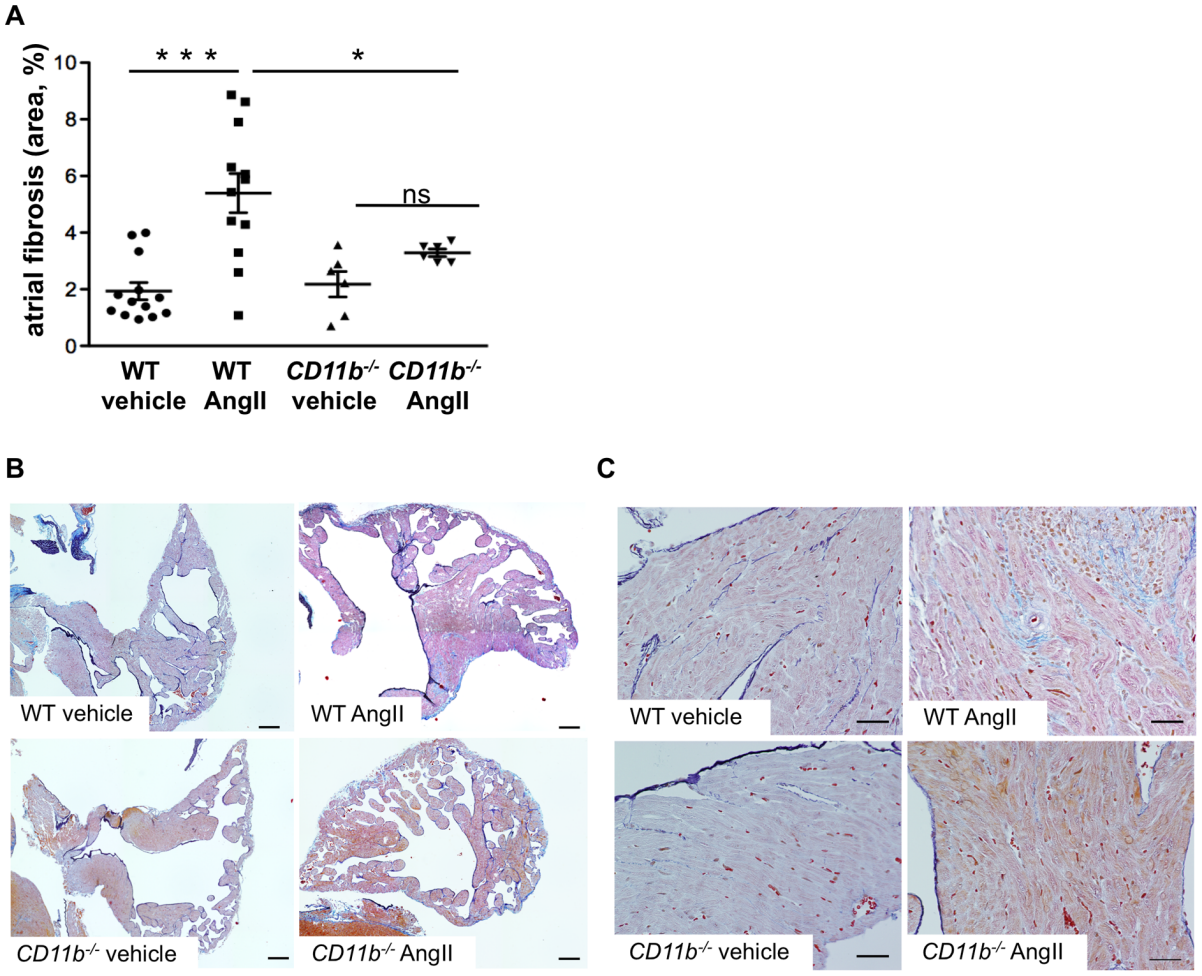
Angiotensin II-induced atrial fibrosis was reduced in CD11b^−/−^ mice. **(A)** Percentage of fibrotic area in atrial sections of WT and CD11b^−/−^ mice upon vehicle or Ang II treatment. *  =  p<0.05, ***  =  p<0.001. **(B, C)** Representative images of Trichrome stained atrial sections with fibrotic tissue stained in light blue merged from 6 individual images with 10× magnification **(B)**, scale bar  =  200 µm and with 40× magnification **(C)**, scale bar  =  40 µm.

To investigate whether increased atrial PMN infiltration and enhanced cardiac MPO deposition translates into aggravated atrial remodeling, we analyzed atrial fibrosis in WT and CD11b^−/−^ mice. Evidenced by increased deposition of matrix proteins, Ang II treatment resulted in profoundly augmented atrial fibrosis compared to vehicle treated WT mice (n = 13, 12; p<0.001). Remarkably, the genesis of atrial fibrosis in Ang II treated CD11b^−/−^ mice was blunted (WT Ang II vs. CD11b^−/−^ Ang II p<0.05) and not different if compared to vehicle-treated CD11b^−/−^ mice (n = 6; p = 0.24; **Fig. 2A–C**).

**Figure 3:**
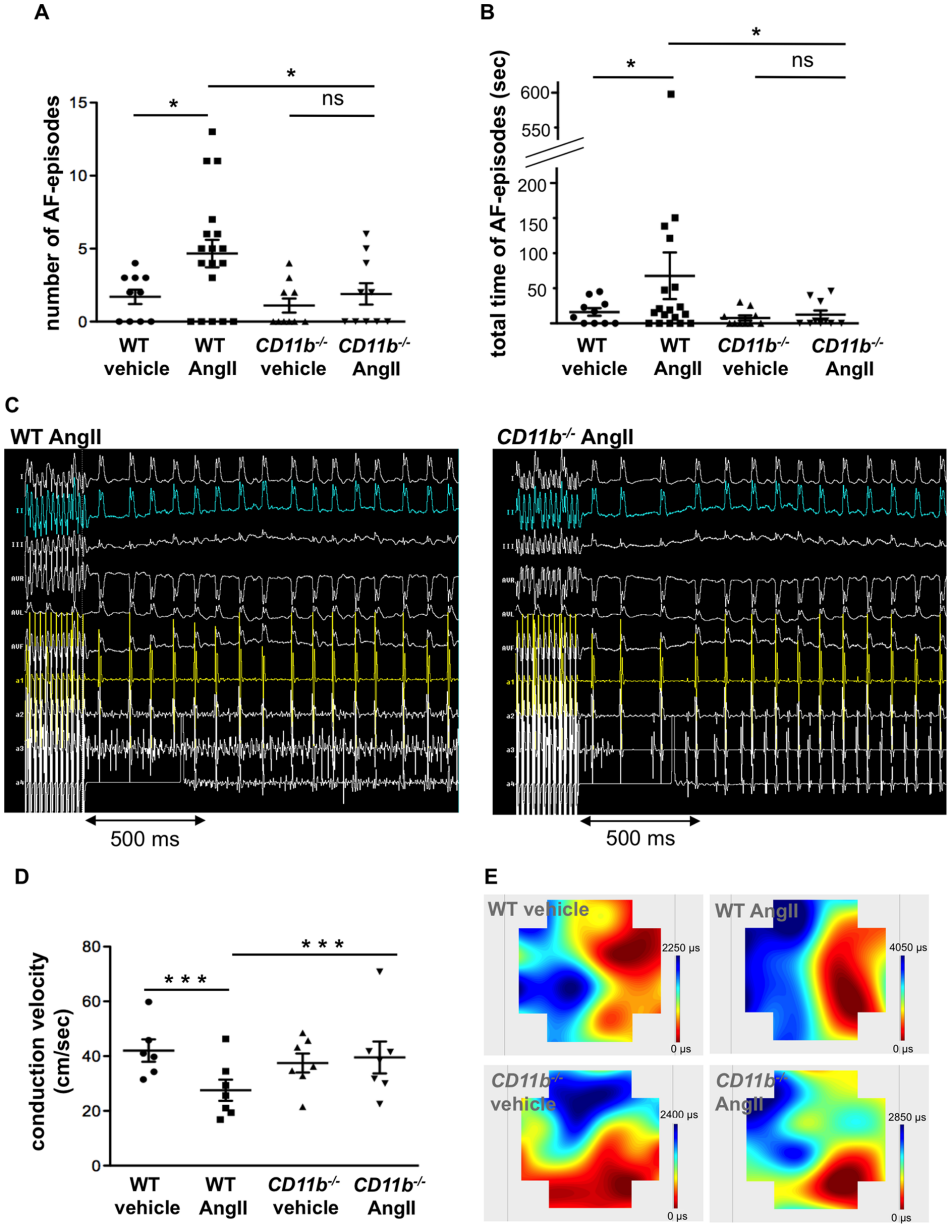
CD11b-deficiency diminished AF vulnerability and preserved conduction velocity following angiotensin II treatment. (A, B) Number and total time of AF-episodes during an electrophysiological stimulation procedure in WT and CD11b^−/−^ mice upon vehicle or Ang II application. *  =  p<0.05. (C) Example electrical tracings of surface and intracardiac leads from Ang II treated WT and CD11b^−/−^ mice during electrophysiological burst stimulation with cycle length of 20 ms. (D) Electrical conduction velocity in propagation direction as assessed by epicardial mapping of Langendorff-perfused hearts of WT and CD11b^−/−^ mice. ***  =  p<0.001. (E) Representative examples of conduction properties of epicardial activation mapping.

Next, we tested whether increased presence of PMN and aggravated atrial fibrosis translate into a lower threshold for initiation of atrial fibrillation. Therefore, we performed *in-vivo* electrophysiological investigations as described previously [37]. Upon controlled local right atrial burst stimulation, inducibility and length of atrial fibrillation was captured. As shown in Fig. 3 WT mice exposed to Ang II (n = 18) revealed markedly increased vulnerability to AF: Number of AF episodes as well as the length of AF episodes were significantly increased as compared to vehicle treated animals (n = 10; p<0.05). In contrast, CD11b^−/−^ mice were protected from the AF-provoking effect of Ang II (n = 8, 12; WT Ang II vs. CD11b^−/−^ Ang II p<0.05) (**Fig. 3** **A–C**). In line with this, P-wave duration was prolonged in Ang II treated WT mice in contrast to CD11b^−/−^ mice (p<0.01) (**Table 1**). In support of these results from electrophysiological investigations, epicardial mapping analyses revealed, that electrical conduction velocity was decreased following chronic Ang II infusion in WT mice (n = 7, 6; Ang II vs. vehicle p<0.001). This deceleration was blunted in CD11b^−/−^ mice (p = 0.6; n = 7; Ang II WT vs. CD11b^−/−^ p<0.001) (**Fig. 3D, E**).

**Table 1:** Electrophysiological parameters derived from surface ECGs.

	WT vehicle	WT Ang II	*CD11b* ^−*/*−^ vehicle	*CD11b* ^−*/*−^ Ang II	P-value
P (ms)	12.6±0.4*	14.1±0.3	12.3±0.3^#^	12.8±0.5^‡^	*0.003; ^#^<0.001; ^‡^0.009 vs. WT Ang II
ARP (ms)	37±5.4	38.8±2.3	37.8±3.2	43.7±3.2	n.s.
QRS (ms)	12.6±0.5	13.2±0.4	12.4±0.3	12.5±0.4	n.s.
QTc (ms)	153.8±8.1	156.2±5.5	158.3±3.0	143.3±5.9	n.s.
HR (bpm)	334±14	345±11	330±12	327±14	n.s.

P, P-wave duration; ARP, atrial refractory period; QRS, QRS duration; QTc, QT interval corrected for heart rate; HR, heart rate; bpm, beats per minute; n.s., not significant.

**Figure 4:**
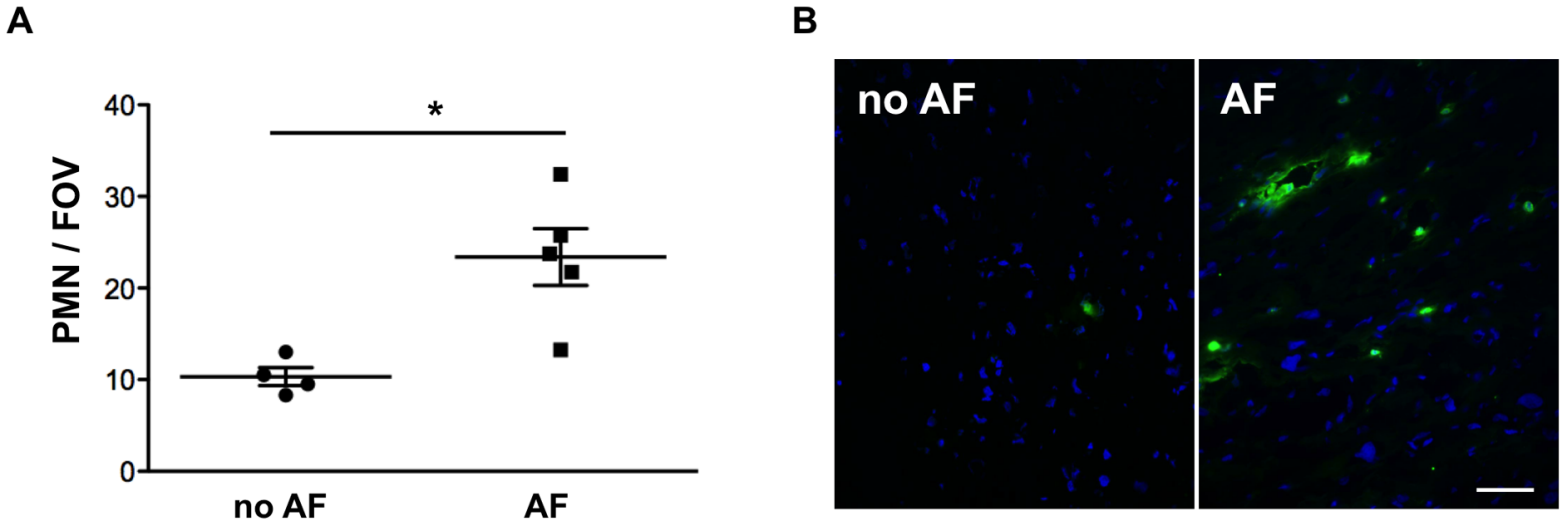
Atrial PMN-infiltration was enhanced in patients with AF. **(A)** Number of MPO-positive leukocytes in sections of atrial appendages of control subjects and patients with persistent AF (FOV  =  field of view, ×40). **(B)** Representative images of immunoreactivity for MPO (green) in human atrial appendage sections. Scale bar  =  15 µm.

Finally, we determined the amount of MPO-positive leukocytes in right atrial appendage tissue of patients with persistent AF (n = 5) or without AF (n = 4), which revealed a significantly increased number of leukocytes with enhanced MPO-deposition in sections of patients with AF as compared to control subjects (p<0.05) (**Fig. 4A, B**).

## Discussion

In the current study we revisited the biological significance of CD11b/CD18 integrin-dependent cardiac recruitment of PMN for atrial fibrosis and AF.

We have reported recently that MPO, stored in primary granules of PMN and released by the cells upon activation, links atrial fibrosis and the susceptibility for atrial fibrillation [25]. However, whether leukocytes are critical for the local distribution of MPO into the tissue has not been answered so far. The current data now show that reduced PMN infiltration in CD11b^−/−^ mice indeed is firmly connected to atrial remodeling and inducibility of AF. Whereas WT mice exposed to 14 days of Ang II infusion exhibited increased atrial fibrosis and concomitantly were noted for decreased threshold for atrial fibrillation, CD11b^−/−^ mice not only displayed attenuated atrial PMN accumulation after Ang II treatment, but also less fibrosis, which translated into reduced susceptibility to atrial fibrillation. Mechanistically, CD11b/CD18 integrin knockout has repeatedly been shown to prevent local PMN extravasation [36], [39], [40], implying a direct negative effect of attenuated atrial PMN infiltration on atrial remodeling. Other potential mechanisms include impaired leukocyte - extracellular matrix interactions [41] and diminished responses of other CD11b expressing cells like macrophages and natural killer cells. Interestingly, a more recent study also supports CD11b-dependent internalization of MPO by endothelial cells [35], thereby providing an additional pathobiological mechanism for a decreased inflammatory atrial milieu in CD11b^−/−^ mice, independently of PMN infiltration itself.

The current study now expands our understanding on the basic mechanisms linking inflammation, atrial remodeling and the development of atrial fibrillation in an important way: The current data reveal that leukocytes, in particular PMN are not only bystanders of AF but at best function as circulating carriers of effector proteins, which then propagate atrial remodeling. Moreover, our results reveal that intimate contact of PMN with the atrial vasculature and PMN recruitment into atrial tissue represent relevant components of atrial fibrosis. Whereas this is accompanied with release of MPO, the enzyme is most probably not the exclusive effector, by which PMN increase the burden of fibrosis in the atria. Superoxide, generated by the celĺs NADPH oxidase, by uncoupled NO-synthases or released by mitochondria is closely linked to the initiation of fibrosis and AF [42], [43]. However the contribution of leukocytes as critical effectors in the pathophysiology of AF has probably been underestimated so far.

Angiotensin II is appreciated as a central effector peptide allowing for atrial remodeling and ultimately the induction of AF [44]. However, these proarrhythmic effects were mainly attributed to the local, myocyte-directed effects of Ang II yielding increased superoxide generation, matrix production and cellular hypertrophy. Interestingly, acute Ang II-mediated proarrhythmic effects in a rat model of ventricular arrhythmia were shown to be dependent on the presence of an aged and more fibrotic myocardium rather than on the occurrence of Ang II induced early afterdepolarisations alone [45]. Given that Ang II-mediated leukocyte activation in the absence of CD11b/CD18-integrins exerted only a slight proarrhythmic effect suggests, that the cytokine-like, leukocyte-activating properties of this peptide contribute to its arrhythmogenicity. Certainly, this does not necessarily imply that inhibition of Ang II-signaling is beneficial in the prevention or therapy of AF, as PMN can be activated by various other stimuli. In fact, inflammatory markers like high-sensitive CRP (hsCRP) and IL-6 are elevated in patients with recurrent AF in an early non-permanent stage of AF [46], but these particular patients did not benefit from Ang II receptor inhibition [47]. However, meta-analyses show an overall beneficial effect for Angiotensin-converting enzyme (ACE) inhibitors and angiotensin receptor blockers (ARBs) in primary and secondary prevention of AF [48], [49], especially in patients already suffering from recurrent AF or with concomitant diseases like hypertension or heart failure. Given the increasing effects of ACE inhibitors and ARBs in patients with exaggerated disease in humans, augmented atrial fibrosis is most likely the result of a variety of pathways, with one of them being mediated by PMN.

Limitations of the current study arise from the fact that we only studied rodents and do not provide data helping to translate the current results into a clinical setting. Furthermore, we investigated AF, which was induced by electrical stimulation instead of detecting spontaneous occurrence of the arrhythmia, e.g. in an ageing cohort of animals.

However, the data clearly reveal the significance of CD11b/CD18 integrins for the initiation and perpetuation of AF, furthermore underscore the role of fibrosis for this disease and call for a more in-depth evaluation of inflammatory mechanisms underlying AF in human pathophysiology.
